# Supplementary material for: Manipulations of multi-frequency waves and signals via multi-partition asynchronous space-time-coding digital metasurface
Source: Nat Commun. 2023 Sep 4;14:5377. doi: 10.1038/s41467-023-41031-0 (PMC10477258; doi:10.1038/s41467-023-41031-0)
Supplement: Supplementary file 1 — Supplementary Information [file 41467_2023_41031_MOESM1_ESM.pdf]

Supplementary Information for

Manipulations of Multi-Frequency Waves and Signals via Multi-Partition Asynchronous Space-Time-Coding Digital Metasurface

Si Ran Wang[1,2,3,5], Jun Yan Dai[1,2,3,5], Qun Yan Zhou[1,2,3], Jun Chen, Ke[1,2,3], Qiang Cheng[1,2,3,*], and Tie Jun Cui[1,2,3,4,*]

[1] *State Key Laboratory of Millimeter Waves, Southeast University, Nanjing 210096, China*
[2] *Institute of Electromagnetic Space, Southeast University, Nanjing 210096, China*
[3] *Frontiers Science Center for Mobile Information Communication and Security, Southeast University, Nanjing 210096, China*
[4] *Pazhou Laboratory, Huangpu, Guangzhou 510555, China*
[5]*These authors contributed equally: Si Ran Wang, Jun Yan Dai*

E-mail: qiangcheng@seu.edu.cn; tjcui@seu.edu.cn

Supplementary Note 1. Selection of the Duty Ratios and Time Delays for Dual-Frequency Manipulations

The selection of M_1, M_2, Δt_1, and Δt_2 for particular amplitudes and phases of the harmonics is as follows:

As derived in the context, the amplitudes and phases of the kth-order harmonic in the partitions 1# and 2# can be written as A_k^1, φ_k^1, A_k^2, φ_k^2, where the subscript and superscript stand for the harmonic and partition order, respectively. For simplicity, we consider the 1st-order generation in partition 1#:

$$\begin{cases} A_1^1 = 2 \cdot M_1 \cdot \left|\frac{\sin \pi M_1}{\pi M_1}\right| \\ \varphi_1^1 = -\frac{\pi}{2}\left[1-(-1)^{\lfloor M_1 \rfloor}\right] \end{cases}. \tag{S1}$$

where $\lfloor\,\rfloor$ indicates the operation of rounding down. If we want to get the desired amplitude and phase (A_1^1 and φ_1^1) for the 1st-order harmonic, we need to solve this nonlinear equation with the numerical method.

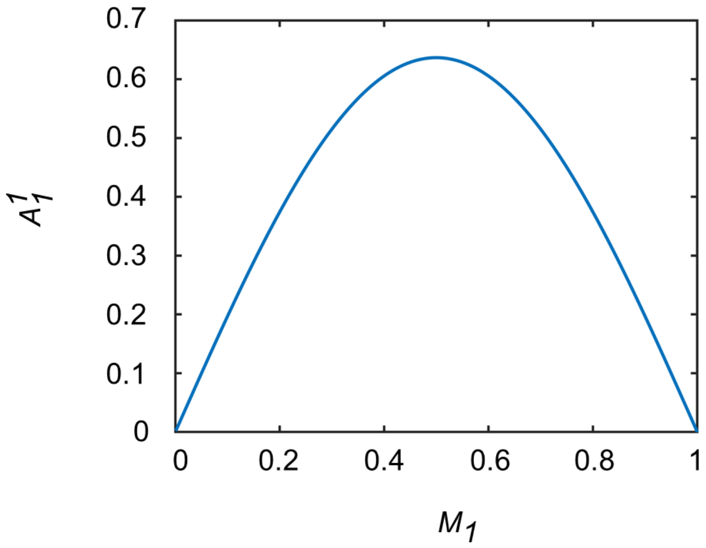

Fig. S1. The relationship between M_1 and the 1st-order harmonic amplitude A_1^1 in partition 1#.

Firstly, from Fig. S1, we can give the relationship between A_1^1 and M_1. From this figure, we can easily get a proper candidate of M_1 for specific amplitude A_1^1.

Secondly, we can get the phase φ_1^1 from Eq. (S1) when M_1 is chosen. If there is a deviation $\Delta\varphi$ between the obtained and desired phases, we can use the time delay Δt_1 for compensation:

$$\Delta t_1 = \frac{-\Delta\varphi}{2\pi f_1}, \tag{S2}$$

in which f_1 represents the modulation frequency of partition 1#.

By following the above two steps, we can get the values of M_1 and Δt_1 to obtain the desired harmonic amplitude and phase in partition 1#. The same approach can be used to determine M_2 and Δt_2 for arbitrary A_k^2, φ_k^2 in partition 2#.

Supplementary Note 2. Scattering Patterns of Dual Frequency Partitions with Different Partition Strategies

To illustrate the influences on scattering patterns using different partitioning strategies, the dark green and orange regions in Fig. S2 are modulated by the frequencies of 100 kHz and 200 kHz, respectively, and the metasurface is illuminated by a plane wave at 4.25 GHz. As a result, the echo frequencies from the dark green and orange regions are 4.2501 GHz and 4.2502 GHz, respectively. According to the generalized Snell's law, a phase gradient should be introduced among the dark green and orange columns to control the scattering beam angle. In Figs. 3(a) and (b), the phase distributions from Columns 1 to 16 are 0, $\pi/2$, π, $3\pi/2$, 0, $\pi/2$, π, $3\pi/2$, $3\pi/2$, π, $\pi/2$, 0, $3\pi/2$, π, $\pi/2$, and 0. We consider two partitioning strategies which are displayed in Figs. S2 (a-b) and Figs. S2 (c-d) respectively. For the first partitioning strategy, the main lobes of scattering patterns point at -44° and +44°, respectively, as shown in Figs. S2 (a-b). For the second partitioning strategy, the main lobes of scattering patterns point at -21° and +21°, respectively, as shown in Figs. S2 (a-b). It should be clarified the scattering patterns were measured when the dual partitions operated alternatively.

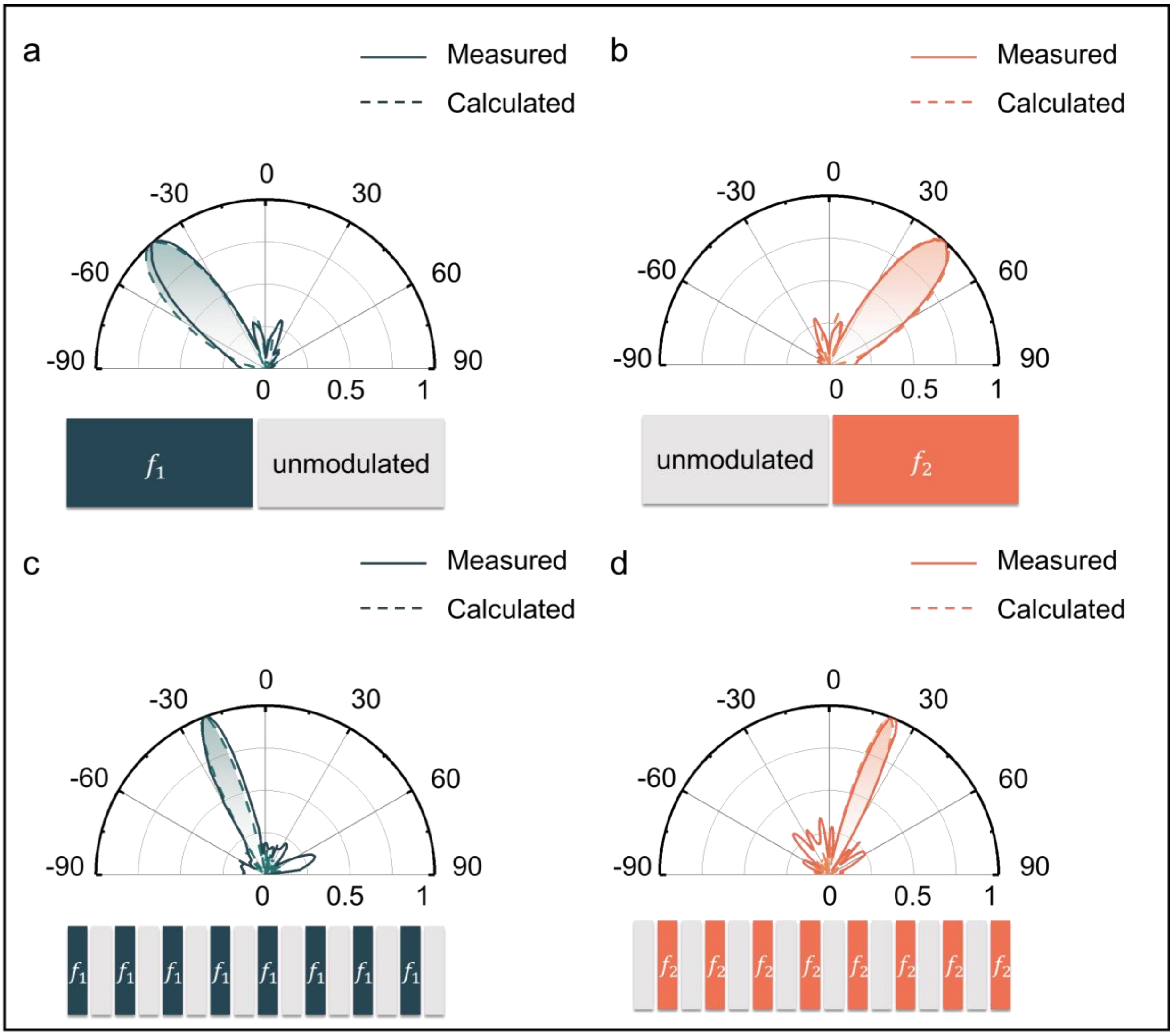

Fig. S2. Measured and calculated scattering patterns from the two partitions at 4.2501 GHz and 4.2502 GHz, respectively. (a) The main lobe of the scattering pattern from the f_1 partition is directed to -44°. (b) The main lobe of the scattering pattern from the f_2 partition is directed to 44°. (c) The main lobe of the scattering pattern from the f_1 partition is directed to -21°. (d) The main lobe of the scattering pattern from the f_2 partition is directed to 21°.

Supplementary Note 3. Simulation Results of the Reflectivity of the Meta-Atom

As illustrated in Figs. S3(a) and (b), we provide the simulated reflection amplitude and phase spectra of the ASTCM element ranging from 2 GHz to 6 GHz based on CST Microwave Studio 2016. It can be observed that the reflection amplitudes under different biasing voltages are greater than -4 dB while the reflection phase range is beyond 2π within (4.25±0.1) GHz.

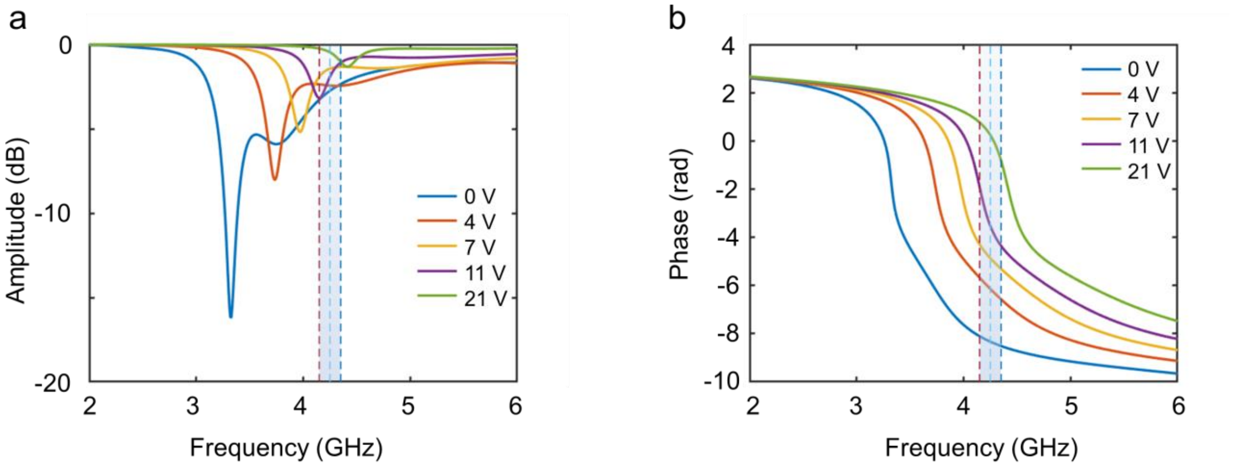

Fig. S3. Reflection amplitude and phase spectra of the meta-atom at different biasing voltages. (a) Reflection amplitude spectrum. (b) Reflection phase spectrum.

Supplementary Note 4. Measurements of the Signal-to-Noise Ratios for Wireless Communications with Different Metasurface Sizes

We measured the signal-to-noise ratio (SNR) when the entire metasurface, two meta-columns, and a single meta-column are employed to transmit one data stream, respectively. Fig. S4 provides the experiment setup, where the ASTCM was illuminated by the transmitting horn connected to a microwave signal generator (Keysight E8267D). The incident frequency was set to 4.25 GHz. The ASTCM was modulated with the controlling platform (PXIe-1082, NI Corp.), which consists of a high-speed I/O bus controller, an FPGA module, a digital-analog conversion module, a DC power supply module, and a timing module, which enables us to provide high-quality biasing signals on the embedded varactor diodes within the metasurface. At the receiving end, the receiving antenna was employed to record the echo waves from the metasurface with a software-defined radio transceiver (NI USRP RIO 2943R).

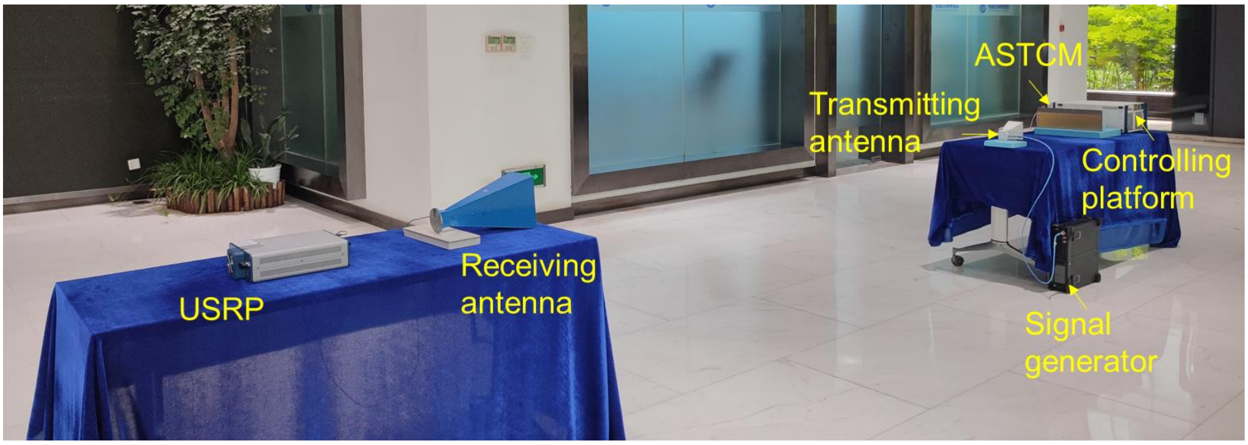

Fig. S4. Experimental scenario of SNR measurement

The SNR results are 41.69 dB (the entire metasurface), 36.78 dB (two meta-columns), and 32.08 dB (single meta-column), indicating that partitioning the aperture will reduce the SNR as predicted. In practical applications, we can increase the metasurface size and use more columns for one frequency channel to enhance the SNR in wireless communications.

Supplementary Note 5. ASTCM-based down-conversion receiver

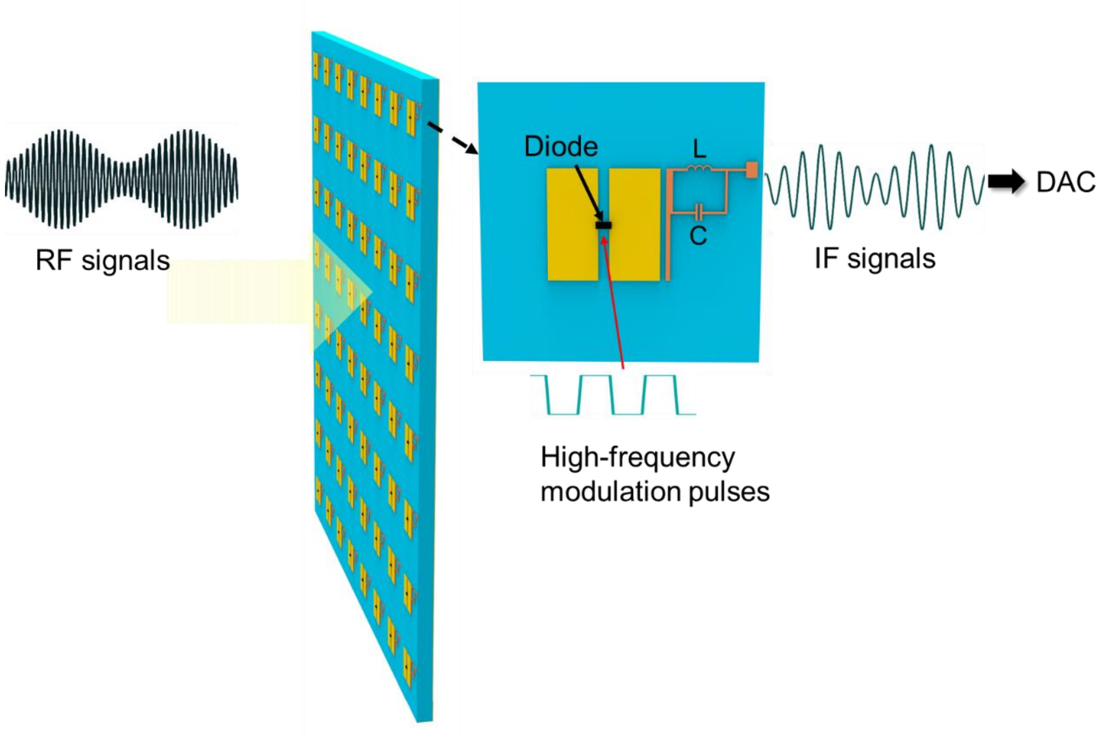

Fig. S5. Diagram of the ASTCM-based down-conversion receiver

As shown in Fig. S5, under the illumination of external RF signals, the diodes of the meta-atoms are biased by periodic voltage waveforms. The impedance of meta-atom becomes a function of the modulation frequency and the voltage Z(f). Thereby the reflection coefficient of the meta-atom can be written as:

$$\Gamma(f) = \frac{Z(f) - Z_0}{Z(f) + Z_0}, \tag{S3}$$

where Z_0 is free-space wave impedance. Then the reflected electric field $\mathbf{E}_r$ becomes:

$$\mathbf{E}_r = \mathbf{E}_i \cdot \Gamma(f). \tag{S4}$$

It has been proved that the spectrum of the reflected field can be manipulated by changing the periodic modulation waveform of the meta-atom. That is to say, we can realize up-conversion or down-conversion of the incident field $\mathbf{E}_i$ directly on the metasurface without using the traditional RF chain. As illustrated in the inset of Fig. S5, for the metasurface-based receiver, the down-conversion should be employed and the corresponding IF signal will be received by an LC filter near the element. Then the IF signal will be sampled by the ADC under the element and sent to DSP for further postprocessing. In this way, we can get the received signals from each element. When different partitions are used to record the waves from various RF channels, the signals sensed by each meta-atom can be collected efficiently in the baseband.
