# Supplementary material for: Manipulations of multi-frequency waves and signals via multi-partition asynchronous space-time-coding digital metasurface
Source: Nat Commun. 2023 Sep 4;14:5377. doi: 10.1038/s41467-023-41031-0 (PMC10477258; doi:10.1038/s41467-023-41031-0)
Supplement: Supplementary file 3 — Description of additional supplementary files [file 41467_2023_41031_MOESM3_ESM.pdf]

# Description of Additional Supplementary Files Document

**Supplementary Movie 1**

Description: This video records the experiment on multi-channel wireless communication using the partition asynchronous space-time-coding digital metasurface.
